# Genome-wide association analysis reveals quantitative trait loci and candidate genes involved in yield components under multiple field environments in cotton (*Gossypium hirsutum*)

**DOI:** 10.1186/s12870-021-03009-2

**Published:** 2021-05-31

**Authors:** Guozhong Zhu, Sen Hou, Xiaohui Song, Xing Wang, Wei Wang, Quanjia Chen, Wangzhen Guo

**Affiliations:** 1 State Key Laboratory of Crop Genetics and Germplasm Enhancement, Cotton Germplasm Enhancement and Application Engineering Research Center (Ministry of Education), Nanjing Agricultural University, Nanjing, 210095 China; 2 Institute of Agricultural Sciences in Coastal Area of Jiangsu Province, Yancheng, 224002 China; 3 Engineering Research Center for Cotton (the Ministry of Education), Xinjiang Agricultural University, Urumqi, 830052 China

**Keywords:** *Gossypium hirsutum*, Yield components, Genome-wide association study, Quantitative trait loci, Multiple field environments

## Abstract

**Background:**

Numerous quantitative trait loci (QTLs) and candidate genes associated with yield-related traits have been identified in cotton by genome-wide association study (GWAS) analysis. However, most of the phenotypic data were from a single or few environments, and the stable loci remained to be validated under multiple field environments.

**Results:**

Here, 242 upland cotton accessions collected from different origins were continuously investigated for phenotypic data of four main yield components, including boll weight (BW) and lint percentage (LP) under 13 field environments, and boll number per plant (BN) and seed index (SI) under 11 environments. Correlation analysis revealed a positive correlation between BN and LP, BW and SI, while SI had a negative correlation with LP and BN. Genetic analysis indicated that LP had the highest heritability estimates of 94.97%, followed by 92.08% for SI, 86.09% for BW, and 72.92% for BN, indicating LP and SI were more suitable traits for genetic improvement. Based on 56,010 high-quality single nucleotide polymorphisms (SNPs) and GWAS analysis, a total of 95 non-redundant QTLs were identified, including 12 of BN, 23 of BW, 45 of LP, and 33 of SI, respectively. Of them, 10 pairs of homologous QTLs were detected between A and D sub-genomes. We also found that 15 co-located QTLs with more than two traits and 12 high-confidence QTLs were detected under more than six environments, respectively. Further, two NET genes (*GH_A08G0716* and *GH_A08G0783*), located in a novel QTL hotspot (qtl24, qtl25 and qlt26) were predominately expressed in early fiber development stages, exhibited significant correlation with LP and SI. The *GH_A07G1389* in the stable qtl19 region encoded a tetratricopeptide repeat (TPR)-like superfamily protein and was a homologous gene involved in short fiber mutant ligon lintless-y (Li_y_), implying important roles in cotton yield.

**Conclusions:**

The present study provides a foundation for understanding the regulatory mechanisms of yield components and may enhance yield improvement through molecular breeding in cotton.

**Supplementary Information:**

The online version contains supplementary material available at 10.1186/s12870-021-03009-2.

## Background

Cotton is an important commercial crop that provides the most natural fiber globally and is also an important source of edible oil. Of the cultivated cotton species, upland cotton (*Gossypium hirsutum* L.) contributes more than 95% of total cotton production due to its high yield and wide adaptability [1]. Developing high-yielding varieties has been one of the essential targets in cotton breeding. However, the improvement of cotton yield via conventional breeding programs remains low and slow because the narrow genetic background of upland cotton has resulted in breeding bottlenecks [2]. Hence, it is of great significance to explore and pyramid the elite quantitative trait loci (QTLs)/genes related to yield components for improving cotton yield through molecular breeding.

The yield components of cotton mainly include boll number per plant (BN), boll weight (BW), lint percentage (LP), seed index (SI), and lint index (LI), which are quantitatively inherited and are easily influenced by the environment [3]. Several QTLs for cotton yield-related traits have been identified using molecular markers and bi-parental linkage mapping analysis [4, 5]. However, it is challenging to exploit QTLs through markers assisted breeding due to the limited number of markers and the large QTL regions. With the rapid development of high-throughput sequencing technologies and statistical methods, the genetic basis of cotton yield-related traits has been preliminarily revealed. In recent five years, the assembly and improvement of tetraploid cultivated cotton genome significantly accelerated the mapping of genes for the important traits in cotton [6–11]. Based on the reference genome sequence, a large number of QTLs and candidate genes associated with yield-related traits were identified by genome-wide association study (GWAS) analysis [12–15]. The power of genome-wide association analysis is mainly based on four factors: the richness of genetic diversity, the veracity of trait acquisition, marker density, and statistical methods [16]. In most previous studies, due to the experimental design of a single environment and the single-locus GWAS approaches, many stable loci remain to be detected. Multi-environment and multi-locus GWAS coupled with improved experimental design and associated methods may increase efficiency to mine QTLs/genes related to fiber yield traits, which is still challenging in cotton breeding.

In the present study, 242 upland cotton accessions with diverse origins were planted in multiple environments over the years for phenotyping investigation of four main yield components, BW and LP under 13 natural environments, and BN and SI under 11 environments. GWAS analysis was conducted based on a multi-locus random-SNP-effect mixed linear model, and stable QTLs associated with yield components were revealed in multiple environments. Combined with transcriptome analysis, the expression patterns of candidate genes were investigated, and key genes contributing to cotton yield were predicted. The results may be helpful to understand the genetic architecture of yield traits better and provide molecular markers and candidate genes for designing high-yielding cotton lines via molecular breeding.

## Results

### Phenotypic variation of the four yield-related traits

**Fig. 1:**
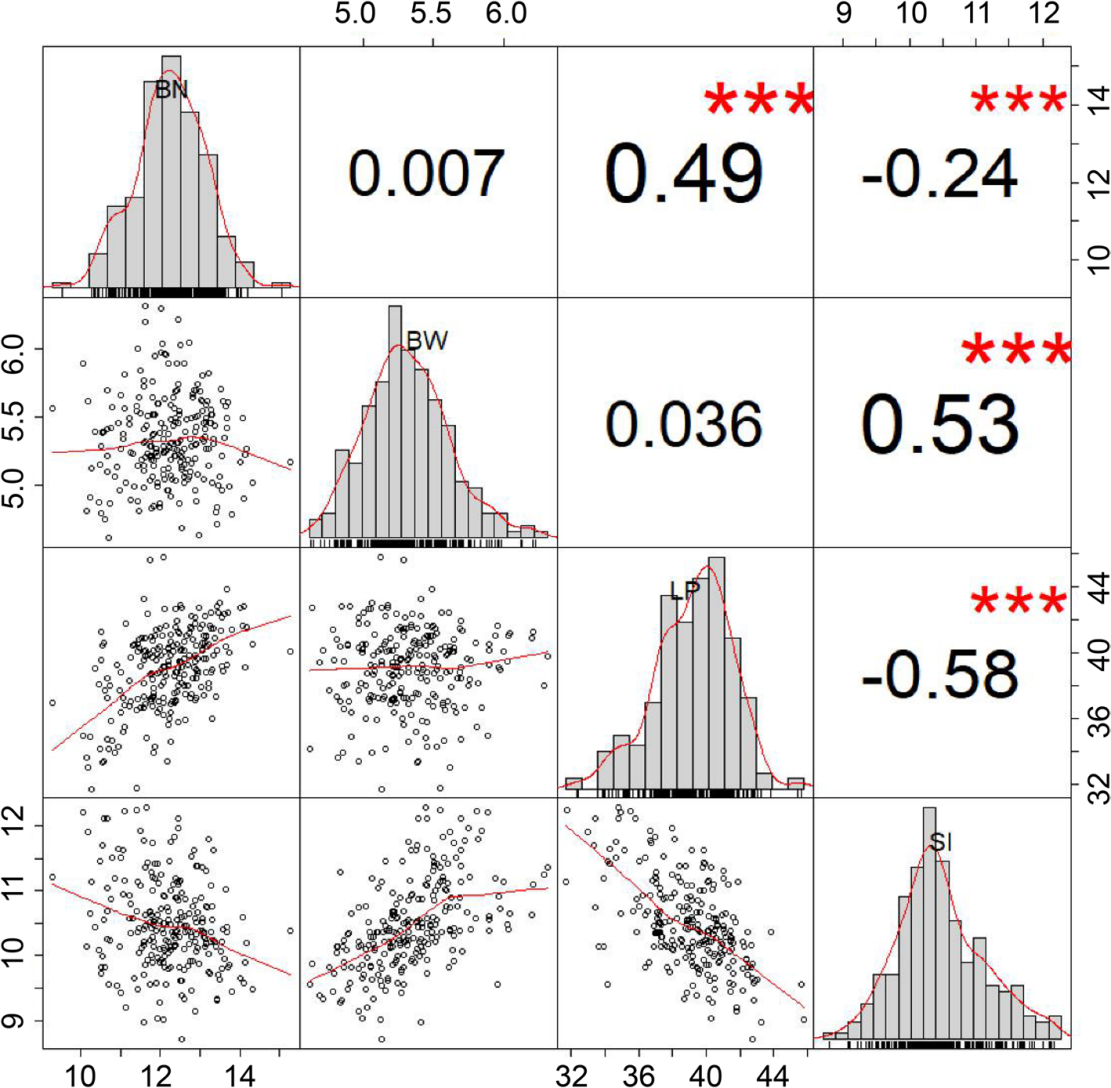
Correlation analysis of four yield-related traits. The number in these boxes indicated correlation coefficient (*R* value). The correlation coefficient and the significance between traits were conducted using BLUP. *** indicated *P* value at the 0.001 levels. BN: boll number per plant; BW: boll weight; LP: lint percentage; SI: seed index

We analyzed the phenotypic data of four yield-related traits boll number (BN), boll weight (BW), lint percentage (LP), and seed index (SI) in multiple field environments to evaluate the phenotypic variation in the natural population of 242 upland cotton accessions (Additional file 1 Table S1). BN, BW, LP, and SI differed significantly from 1.7 to 35.5, 2.5 g to 9.1 g, 23.0% to 50.2%, and 6.7 g to 17.1 g, respectively (Additional file 2 Table S2). BN exhibited the largest coefficient of variation (CV), ranging from 13.13% to 24.26%, while LP showed the smallest CV ranging from 7.19% to 10.53%. The best linear unbiased prediction (BLUP) across multiple environments estimates showed that the phenotypic value of BN, BW, LP and SI from BLUP ranged from 9.3 to 15.3, 4.6 g to 6.3 g, 31.7% to 45.8%, and 8.7 g to 12.3 g, respectively (Additional file 2 Table S2, Additional file 3 Fig. S1). The broad-sense heritability (*h*^*2*^) of each trait was estimated based on multi-environment phenotypic data (Table 1) to explore the breeding potential of the traits. The high heritability estimates were found for LP (94.97%) and SI (92.08%), followed by BW (86.09%). However, the heritability of BN (72.92%) was the lowest, indicating the BN was inclined to be influenced by environments. The analysis of variance (ANOVA) was performed to investigate the effects of genotype (G), environment (E), and G × E interactions among the four traits in multi-environment (Table 1). The results indicated that these traits were also significantly subjected to genotypes and environments interactions. Correlation analysis showed a positive correlation between BN and LP, while SI showed a positive correlation with BW and a negative correlation with BN and LP (Fig. 1).

**Table 1:** The analysis of variance and broad heritability of four yield-related traits

Trait	Factors	Df	Sum-sq	Mean-sq	*F*	*h*^2^(%)
	Env	10	199,757.98	19,975.80	4137.62***	
	Rep(Env)	11	858.47	78.04	16.17***	
BN	Geno	241	8938.48	37.09	7.68***	72.92
	Geno × Env	2405	24,123.73	10.03	2.08***	
	Error	2643	12,760.00	4.83		
	Env	12	1733.65	144.47	717.61***	
	Rep(Env)	13	62.04	4.77	23.7***	
BW	Geno	241	880.89	3.66	18.16***	86.09
	Geno × Env	2886	1475.69	0.51	2.54***	
	Error	3111	626.31	0.20		
	Env	12	28,466.88	2372.24	899.41***	
	Rep(Env)	13	112.00	8.62	3.27***	
LP	Geno	241	39,670.63	164.61	62.41***	94.97
	Geno × Env	2887	24,035.90	8.33	3.16***	
	Error	3108	8197.54	2.64		
	Env	10	3020.86	302.09	892.59***	
	Rep(Env)	11	21.45	1.95	5.76***	
SI	Geno	241	2966.70	12.31	36.37***	92.08
	Geno × Env	2409	2358.90	0.98	2.89***	
	Error	2633	891.11	0.34		

BN: boll number per plant; BW: boll weight; LP: lint percentage; SI: seed index; Env: environment; Rep: replication; Geno: genotype; *** indicated P value at the 0.0001 levels

### Genome-wide association analysis

**Fig. 2:**
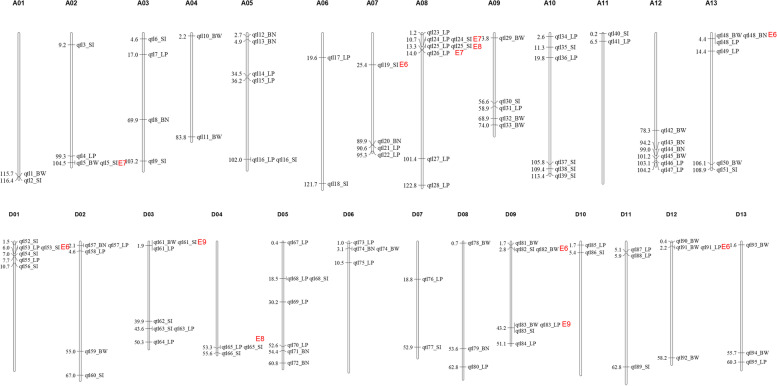
Distributions of candidate QTLs associated with four yield-related traits. The numbers on the left of chromosomes indicates physical distance of the corresponding QTLs. The red character indicates high-confidence QTLs with detected times in different environments (E)

To explore the genetic factors associated with the four yield-related traits, we conducted a GWAS analysis using the genotypic data of 56,010 high-quality SNPs ([17], Additional file 4 Table S3) and the phenotypic data of the four yield-related traits in multiple environments and the BLUP. In total, 560 quantitative trait nucleotides (QTNs) were identified as loci significantly associated with the four traits. With 200 kb as the linkage disequilibrium (LD) threshold for merging QTNs into the same QTL, a total of 360 QTLs of four traits were identified from all environments and BLUP (Additional file 5 Table S4). Further, QTLs consistent across at least two environments were declared as stable QTLs; thus, 95 candidate QTLs were identified with 12 of BN, 23 of BW, 45 of LP and 33 of SI (Fig. 2 and Additional file 6 Table S5). Of them, 38 co-located with previous studies (Additional file 6 Table S5), and 57 were novel and were reported for the first time in the current study. Furthermore, 10 pairs of homologous QTLs were detected between A and D sub-genomes (Additional file 7 Table S6), indicating a common selection of two sub-genomes to improve yield components in upland cotton.

There were 15 QTLs detected simultaneously to be associated with two or more traits (Additional file 6 Table S5, Additional file 8 Fig. S2). For example, nine common QTLs for LP and SI were identified, consistent with the high correlation (R = -0.58) between the two traits. These candidate QTLs were widely distributed on the 26 chromosomes with a little more on At sub-genome comparing to Dt sub-genome (Additional file 9 Fig. S3).

The QTLs consistently detected in more than six environments were defined as high-confidence QTLs; thus, 12 high-confidence QTLs were identified (Fig. 2, Additional file 6 Table S5). For example, qtl24 was associated with LP in seven environments and qtl19 with SI in six environments. Furthermore, three QTLs were found to be associated with three traits simultaneously: qtl48 were identified to co-locate with BW, BN, and LP in six environments; qtl61 and qtl83 with BW, SI, and LP in nine environments, implying the genetic stability of these QTLs for yield components in upland cotton.

### Identification of candidate genes in yield-related QTL regions

**Fig. 3:**
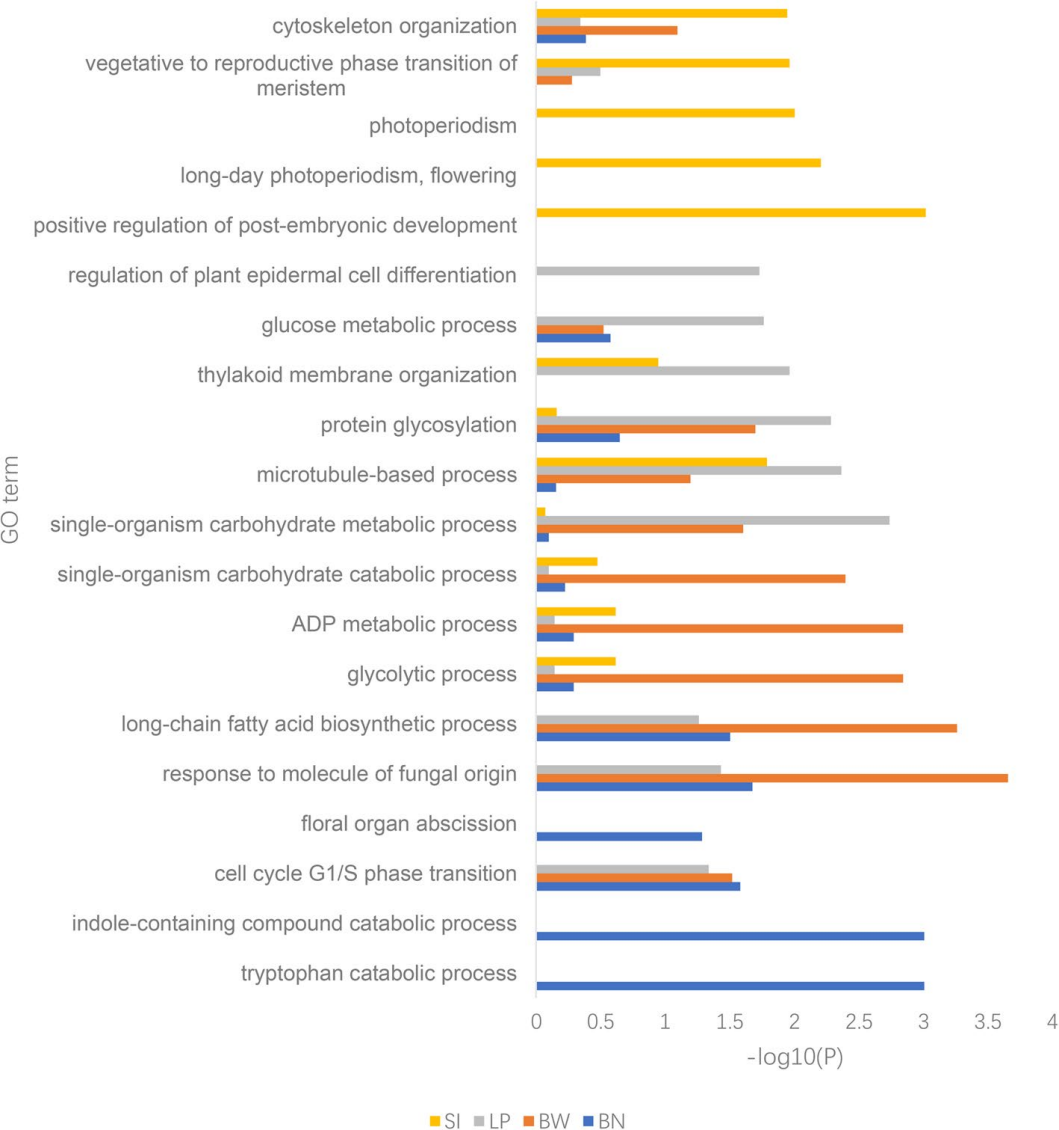
GO analysis of candidate genes associated with four yield-related traits

Genes located in the candidate QTL regions and with an expression of more than 3 transcripts per million (TPM) in cotton tissues were extracted based on the released *G. hirsutum* TM-1 genome [11]. A total of 4144 genes were located in the candidate QTL regions, with 547 related to BN, 1096 to BW, 2236 to LP and 1430 to SI. By filtering the low expressed genes, 1490 candidate genes were identified, with 347 related to BN, 407 to BW, 606 to LP and 424 to SI (Additional file 10 Table S7). Gene Ontology (GO) analysis for each trait was individually conducted to investigate the candidate genes' function (Fig. 3, Additional file 11 Table S8). We found that candidate genes from the QTLs region of different traits were predominately enriched in different biological processes. For LP, the function of genes was mainly enriched in glycol-metabolism-related processes, such as single-organism carbohydrate metabolic process, glucose metabolic process, carbohydrate biosynthetic process. Besides, other processes closely related to fiber development were enriched, such as microtubule-based process, thylakoid membrane organization, regulation of plant epidermal cell differentiation, and plant-type secondary cell wall biogenesis. For SI, embryonic development and flowering-related processes were preferentially enriched, such as positive regulation of post-embryonic development, long-day photoperiodism, flowering, and vegetative to the reproductive phase transition meristem. For BW, the candidate genes involved in energy metabolism, including long-chain fatty acid biosynthetic process, glycolytic process, ADP metabolic process and single-organism carbohydrate catabolic process. For BN, the enriched processes contained tryptophan catabolic process, cell cycle G1/S phase transition and floral organ abscission. In general, these predominately enriched biological processes from QTL regions of each trait were highly related to the developmental process of these yield components.

### Key QTLs and genes associated with yield-related traits

**Fig. 4:**
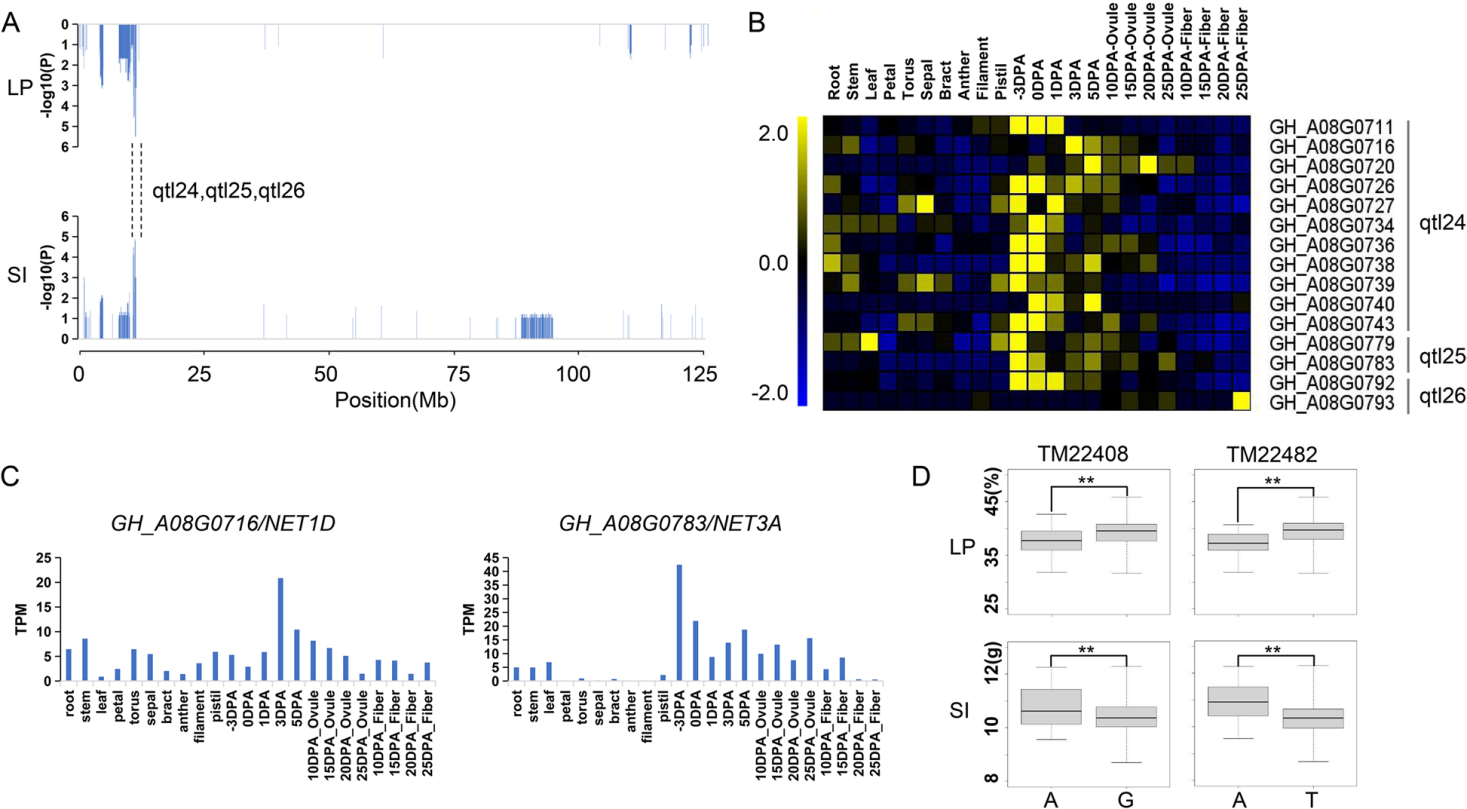
QTLs and candidate genes located on a QTL hotspot on chromosome A08. **a**. The QTL hotspot associated with LP and SI on chromosome A08. **b**. Expression heatmap of candidate genes in the three stable QTLs. **c**. The expression patterns of two NET genes in different tissues. **d**. Box plots for the phenotypic values of QTN closest to the two NET genes

Among 12 high-confidence QTLs, we found a QTL hotspot (A08: 10.7—14.6 Mb) containing three novel QTLs, qtl24, qtl25 and qlt26 for LP, SI and LP, respectively (Fig. 4a). Via tissue and organ transcriptome profiling, we found that 15 genes located on the stable QTL hotspot were predominantly expressed in ovule or fiber during fiber development (Fig. 4b). Of them, two encoding kinases interacting (KIP1-like) family protein genes (*GH_A08G0716* and *GH_A08G0783*), its' orthologous genes encoding plant-specific Networked (NET) superfamily of actin-binding proteins in *Arabidopsis*, were identified in qtl24 and qtl25, respectively. The two genes were highly expressed in fiber development, especially at the early fiber development stages (Fig. 4c). Further, two QTNs (TM22408 and TM22482) which were closest to the two NET genes, respectively, were selected to investigate the correlation between genes and yield components. By comparing the phenotypic difference of the two genotypes (Fig. 4d), the LP values with a G genotype were significantly higher than that with an A genotype in QTN TM22408, while the SI values were lower with a G genotype. In QTN TM22482, a T genotype showed higher LP values and lower SI values than an A genotype. The results indicated that these two genes have potential functions in increasing LP. We also found two genes qtl24: *GH_A08G0734* and qtl26: *GH_A08G0793*, highly expressed at 0 DPA and 25 DPA of fiber development, respectively. *GH_A08G0734* encoded a protein phosphatase 2A (PP2A) regulatory B subunit family protein to regulate the brassinosteroid-mediated signaling pathway [18]. *GH_A08G0793* encoded a bidirectional sugar transporter SWEET12-like protein, which also plays a crucial role in fiber development [19].

Cotton fiber and seed development are equally important because of the specialized hair-like fibers derived from the seed-coat epidermis. Of the stable QTLs, most were simultaneously associated with LP and SI. We found that most candidate genes were highly expressed in ovule at the early stages of fiber development through transcription profile analysis, indicating that SI plays a vital role in fiber yield. A qtl19 was identified to be associated with SI and 17 candidate genes on chromosome A07 in the region of 25.4—26.5 Mb. Of the genes, most were highly expressed from -3 DPA to 1 DPA during fiber development (Additional file 12 Fig. S4a). *GH_A07G1389* encoded a tetratricopeptide repeat (TPR)-like superfamily protein, previously reported to be related to Ligon-lintless mutant phenotype, and was highly expressed from -3 DPA to 1 DPA during fiber development (Additional file 12 Fig. 4b). With the closest QTN TM19889, the LP values with a G genotype were significantly higher than that with an A genotype, while the SI values were lower with a G genotype (Additional file 12 Fig. S4c). We also found that a qtl83 (D09:43.2—44.0 Mb), which was detected in nine different environments, was simultaneously associated with BW, LP and SI. Within the qtl83 region, 28 candidate genes were identified, and most of them were involved in fiber development, such as *GH_D09G1670* (*EXPA4*), *GH_D09G1688* (*AGD7*) and *GH_D09G1611* (*ARF16*), indicating their important roles in yield components formation.

## Discussion

Improving yield is the first breeding objective in crop breeding practices. Cotton is an important economic crop, and its yield components mainly include boll number, boll weight, lint percentage, seed index, lint index etc. It is of great significance to discover QTLs and candidate genes related to yield traits for genetic improvement of cotton to develop high-yielding cultivars. With the release of genome-wide sequence information, lots of QTLs and genes associated with cotton yield traits were identified by GWAS analysis [12, 20–22]. Nevertheless, the yield components are complex quantitative traits controlled by multiple genes and affected by environments. Most GWAS methods are based on a fixed-SNP-effect mixed linear model (MLM) and single-marker analysis, requiring strict correction for the *P* values and containing many minor-effect QTLs. Previous studies using the multi-locus mixed linear model have also indicated that the model can improve the power and robustness of association analysis [23]. In this study, 242 upland cotton accessions with different origins were planted and investigated under multiple field environments over the years. Through GWAS analysis with the multi-locus mixed linear model, stable QTLs and candidate genes for the four main yield components were systematically revealed.

BN is a major yield component that significantly contributes to the individual plant yield of cotton [22]. In a previous study, we have also found that BN is the most important factor in cotton fiber yield [20]. However, the high CV value and low heritability of BN (72.92%) indicate that the trait is greatly influenced by the environment and is difficult to be controlled in genetic improvement. The BW of cotton is a complex trait affected by seed weight and fiber weight per boll; therefore, SI and LP are two important traits in cotton yield improvement. In this study, LP and SI showed high heritability of 94.97% and 92.08%, respectively, similar to previous reports [12, 16].

Furthermore, a significant negative correlation was identified between LP and SI, and many co-located QTLs were identified both for LP and SI [12, 22]. At present, most of the studies focus on genetic improvement of LP [12, 21], while SI is relatively few. We found that there was potential to improve fiber yield through phenotypic analysis by balancing SI and LP values of cotton. As the carrier of fiber, the ovule plays an essential role in fiber development [24]. Manipulating some of the candidate genes identified in fiber cells has yielded no effect or only a marginally positive effect on fiber yield or quality [25], implying the synergistic effect between ovule and fiber development in contributing cotton yield.

We found that many fiber development candidate genes were highly expressed in ovules. During the evolution and domestication of cotton, cotton fiber yield has been strongly correlated with increased seed size [26, 27]. However, the negative correlation between SI and LP indicated that the small seed was conducive to increasing cotton fiber yield for modern cultivars. Therefore, we speculate that a suitable range of seed size effectively increases cotton fiber yield and keeps seed vigor. The regulation of ovule on fiber development could be further explored to identify candidate genes for fiber yield improvement.

The previous studies have detected a large number of associated loci for fiber yield via GWAS analysis. However, the yield traits are quantitatively inherited and easily affected by environmental factors [28]. Thus, most detected QTLs are difficult to confirm and limited in use. In the present study, 560 QTNs were identified. However, several QTNs had minimal QTN effect and almost zero of *r*^*2*^, which might be false positive from the software algorithm and were useless for breeding. We also found that these QTNs with low *r*^*2*^ values were associated only in a single environment. Therefore, to improve the authenticity and validity of QTLs, we selected QTLs detected in two or more environments as candidate QTLs. In the 95 candidate QTLs, 38 co-located QTLs have been identified in multiple reported studies [12, 13, 15, 20, 21, 23, 29, 30]. In addition, 57 novel QTLs were detected in multiple environments; for example, qtl83 was associated with BW, LP and SI in nine environments, providing more loci and markers for genetic improvement of cotton yield traits. We also found that most of the candidate QTLs were trait-specific, implying the independent regulation mechanism among different yield components. However, 15 QTLs were identified simultaneously in multiple traits and contributed pleiotropically to the improvement of cotton yield through marker-assisted breeding.

To identify key genes suitable for breeding utilization of cotton, we selected the 12 high-confidence QTLs detected in multiple environments for further analysis. In these QTLs, a QTL hotspot contained three novel QTLs (qtl24, qtl25 and qlt26) on chromosome A08 was detected. The qtl25 explained the highest phenotypic variation with 26.49% for LP and 20.25% for SI. In the QTL hotspot, 15 candidate genes were identified, and two genes (*GH_A08G0716* and *GH_A08G0783*) encoding the NET superfamily proteins, which potentially couples different membranes to the actin cytoskeleton in plant cells, were further analyzed. In *Arabidopsis*, NET1A is anchored at the plasma membrane and predominates at cell junctions, the plasmodesmata [31]. Besides, NET1A was also found to be involved in response to abscisic acid (ABA), the mitogen-activated protein kinase (MAPK) signaling pathway, and the calcium transduction pathway in upland cotton [32]. The two NET genes identified in this study might play an important role during fiber development. SI was also a crucial trait for cotton yield. The qtl19 was only associated with SI in six different environments. Within this QTL region, *GH_A07G1389* encoded a tetratricopeptide repeat (TPR)-like superfamily protein, and the gene was a homologous gene of a reported gene that was responsible for the Li_y_ short fiber phenotype. Silencing of the *Li*_*y*_ gene could significantly reduce the fiber length in upland cotton [33]. *GH_A07G1389* showed a high expression from -3 DPA to 1 DPA in fiber development, inferring that the differential expression of this gene in ovules might affect the fiber development of cotton. A large number of fiber development-related genes in the stable QTL interval were also found, such as *GH_A08G0793* (*SWEET12*) [18] and *GH_D09G1670* (*EXPA4*) [34], which might contribute to fiber yield improvement in breeding practice.

## Conclusions

Mining the elite loci and alleles related to yield components has more practical value for genetic improvement of crop yield via molecular breeding by design. Here, the phenotyping data of four main yield components, boll number per plant (BN), boll weight (BW), lint percentage (LP) and seed index (SI), from 242 upland cotton accessions were continuously investigated under multiple field environments over the years. Genetic analysis indicated that LP had the highest heritability, following SI, BW, and BN. Through GWAS analysis, we identified 95 non-redundant and stable QTLs with 12, 23, 45, 33 for BN, BW, LP and SI, respectively. Of them, 15 QTLs were associated simultaneously with two or more traits and contributed pleiotropically cotton yield components, and 12 QTLs were detected repeatedly in more than six environments as high-confidence QTLs. Also, 10 pairs of homologous QTLs between A and D sub-genomes were detected. Further, we identified two NET genes (*GH_A08G0716* and *GH_A08G0783*) located in a stable QTL hotspot (qtl24, qtl25 and qlt26) and predominately expressed in early fiber development stages. A gene encoding tetratricopeptide repeat (TPR)-like superfamily protein (*GH_A07G1389*) in stable qtl19 region, were important candidate genes for improving the cotton yield. The present study might contribute novel elite loci and gene resources for yield improvement in cotton breeding practice.

## Methods

### Plant Materials

A natural population of 242 upland cotton accessions (Additional file 1 Table S1) was planted in 13 natural environments following local agronomic practices in China, including Korla, Xinxiang and Nanyang in 2011, 2012 and 2013, Shawan in 2016 and 2017, Yancheng and Dangtu in 2018 (Additional file 2 Table S2). All the accessions were planted with a random complete block design (RCBD) with two replicates in every environment and two rows in each replicate. In korla and Shawan, row length was 2 m, with a 66 + 10 cm (wide/narrow) row distance and a 10 cm plant distance. Row length was 5 m in Xinxiang, and row distance was 80 cm with a 25 cm plant distance. In Nanyang, Yancheng and Dangtu, row length was 4.5 m, row distance was 80 cm and plant distance was 33 cm. All accessions were collected and preserved by Nanjing Agricultural University, China. All necessary permits for planting and investigating the set of the natural population were obtained from Nanjing Agricultural University, China.

### Phenotype investigation and data analysis

Four yield-related traits BN, BW, LP and SI were measured in each environment to explore the phenotypic variation of cotton. At the mature stage of cotton, ten plants for each accession were selected randomly from the middle of each row. The BN was counted with ten biological replicates of each accession. The BW was weighed with 20 mature boll samples randomly harvested from the middle branches with 2 bolls per plant. After ginning, the LP was calculated based on the fraction of lint weight to seed-cotton weight, and the SI was weighed with 100 normally developed seeds.

In this study, the data for BW and LP from 13 natural environments, and BN and SI from 11 natural environments, were used for analysis. The best linear unbiased predictors (BLUPs) of the genotypic effects based on a mixed linear model for each trait were conducted by the lme4 package of R software [35]. The breeding value was evaluated by the best linear unbiased predictors adding population mean (marked as “BLUP”) and used for correlation analysis and GWAS. The density distributions of phenotypic value were performed using “ggplot2” package in R software. ANOVA was conducted for phenotypic data in different environments using PROC GLM procedure in SAS software [36]. All variations from different sources were treated as random effects with a multi environments random blocks linear model. The statistical model was y_ijk_ = μ + α_i_ + β_j_ + γ_kj_ + (αβ) _ij_ + ε_ijk_, that μ indicates the overall mean, α_i_ indicates the genetic effect of the i_th_ genotype, β_j_ indicates the effect of the j_th_ environment, γ_kj_ indicates the random effect of the k_th_ replicate in the j_th_ environment, (αβ)_ij_ indicates the interaction effect between the i_th_ genotype and the j_th_ environment, ε_ijk_ indicates the residual [37]. The formula calculated the broad-sense heritability of each trait: *h*^*2*^ = *σ*^*2*^_*g*_*/(σ*^*2*^_*g*_ + *σ*^*2*^_*ge*_*/n* + *σ*^*2*^_*ε*_*/rn)*, that *σ*^*2*^_*g*_ indicates the genotype variance, *σ*^*2*^_*ge*_ indicates the genotype by environment interaction variance, *σ*^*2*^_*ε*_ indicates the error variance, *n* indicates the number of environments, and *r* indicates the number of replications [38]. Variance values used for broad-sense heritability were calculated using the REML method for the SAS VARCOMP procedure in SAS software. The correlation coefficient and the significance between traits were conducted by SPSS 22.0 software.

### SNP genotyping

Genomic DNA of young leaf tissues of each accession was extracted using the CTAB method as described by Paterson et al. (1993) [39]. The 242 accessions were genotyped by the CottonSNP80K array, which contained 77,774 SNPs [16]. After filtering the SNPs with a calling rate of < 0.9 and MAF < 0.05, high-quality SNPs were obtained. The probe sequences of the high-quality SNPs were mapped to the TM-1 V2.1 genome [11] to determine the exact physical location. When a probe sequence is mapped to multiple loci, the corresponding SNP was discarded. Finally, a dataset that contained 56,010 unique mapped high-quality SNPs was used for GWAS analysis.

### GWAS analysis

Based on a multi-locus random-SNP-effect mixed linear model (mrMLM) [22], six methods (“mrMLM”, “FASTmrMLM”, “FASTmrEMMA”, “pKWmEB”, “ISIS EM-BLASSO” and “pLARmEB”) in the R package “mrMLM” were used to identify QTNs for each trait. The parameters of program were set as: Critical *P*-value = 0.001; Search radius of candidate gene (Kb) = 100; Critical LOD score = 3. Also, we selected Q + K statistical model for GWAS analysis. A kinship (K) matrix was obtained directly in the “mrMLM” program. According to our previous study [40], k = 3 was selected, and a population structure (Q) matrix was calculated by admixture version 1.3. The 52 data sets, including the phenotyping data from the different environments and the BLUP for each trait, were used for the GWAS analysis.

### Identification of QTLs and candidate genes

Due to the uneven distribution of the genotyped SNPs and referenced in the previous report [19], we selected the lowest LD (200 kb) of the chromosome as a threshold to merge the adjacent QTNs into the same QTL. According to a self-written shell script, candidate genes were obtained from the QTL regions on the reference genome. To determine which genes are related to cotton yield components, the transcriptome FastQ data of TM-1 tissues, including root, stem, leaf, petal, torus, sepal, bract, anther, filament, pistil, ovule and fiber tissues at -3, 0, 1, 3, 5 days post-anthesis (DPA), ovules at 10, 15, 20, and 25 DPA, and fiber tissues at 10, 15, 20, and 25 DPA, were downloaded from NCBI Sequence Read Archive collection PRJNA490626 [11]. Using the mapping software Hisat2 [41], uniquely mapped reads were mapped to the reference genome and counted for all cotton annotated genes using HTSeq-Count [42]. The following parameters were used for the counting process: -f bam, -r name, -s no, -m union, -a 20. These count files were used for the identification of DEGs using the program edgeR [43]. A candidate gene was considered stable when the TPM (transcripts per million) was greater than 3 and was used for further analysis. AgriGO V2.0, an online bioinformatics tool, was used to analyze the candidate genes [44].

## Supplementary Information

**Additional file 1: Table S1.** Information of 242 upland cotton accessions used in this study.

**Additional file 2: Table S2.** Phenotypic variation for four yield-related traits in multiple field environments and BLUP.

**Additional file 3: Figure S1.** Density distributions of four yield-related traits in upland cotton natural population.

**Additional file 4: Table S3.** Information on high quality SNPs genotyped by CottonSNP80K array.

**Additional file 5: Table S4.** QTNs and QTLs of boll number per plant, boll weight, lint percentage and seed index detected by multi-loci MLM model.

**Additional file 6: Table S5.** The candidate QTLs, co-located QTLs and high-confidence QTLs identified for the four yield-related traits.

**Additional file 7: Table S6.** Information of homologous QTLs for the four yield-related traits.

**Additional file 8: Figure S2.** The number of candidate QTLs associated with the yield-related traits on At and Dt sub-genomes.

**Additional file 9: Figure S3.** Venn diagram of QTLs associated with four yield-related traits.

**Additional file 10: Table S7.** Expression profile of candidate genes from the QTL regions related to the four yield-related traits.

**Additional file 11: Table S8.** Function analysis of genes in QTL regions related to the four yield-related traits.

**Additional file 12: Figure S4.** Candidate genes related to the qlt19 associated with SI and located on chromosome A07. **a**. Expression heatmap of candidate genes in qlt19. **b**. The expression pattern of *GH_A07G1389* in different tissues. **c**. Box plots for the phenotypic values of QTN closest to *GH_A07G1389*.

## Data Availability

RNA-Seq data in this study have been deposited at the National Center of Biotechnology Information (NCBI, http://www.ncbi.nlm.nih.gov/) under the accessions PRJNA490626.

## Abbreviations

ANOVA: Analysis of variance
BLUPs: The best linear unbiased predictors
BLUP: The best linear unbiased prediction
BN: Boll number
GO: Gene ontology
GWAS: Genome-wide association studies
SI: Seed index
LD: Linkage disequilibrium
LP: Lint percentage
MAF: Minor allele frequency
QTLs: Quantitative trait loci
QTNs: Quantitative trait nucleotides
BW: Boll weight
SNP: Single nucleotide polymorphism
