# Supplementary figures and images for: Genome-wide association analysis reveals quantitative trait loci and candidate genes involved in yield components under multiple field environments in cotton (Gossypium hirsutum)
Source: BMC Plant Biol. 2021 May 31;21:250. doi: 10.1186/s12870-021-03009-2 (PMC8167989; doi:10.1186/s12870-021-03009-2)

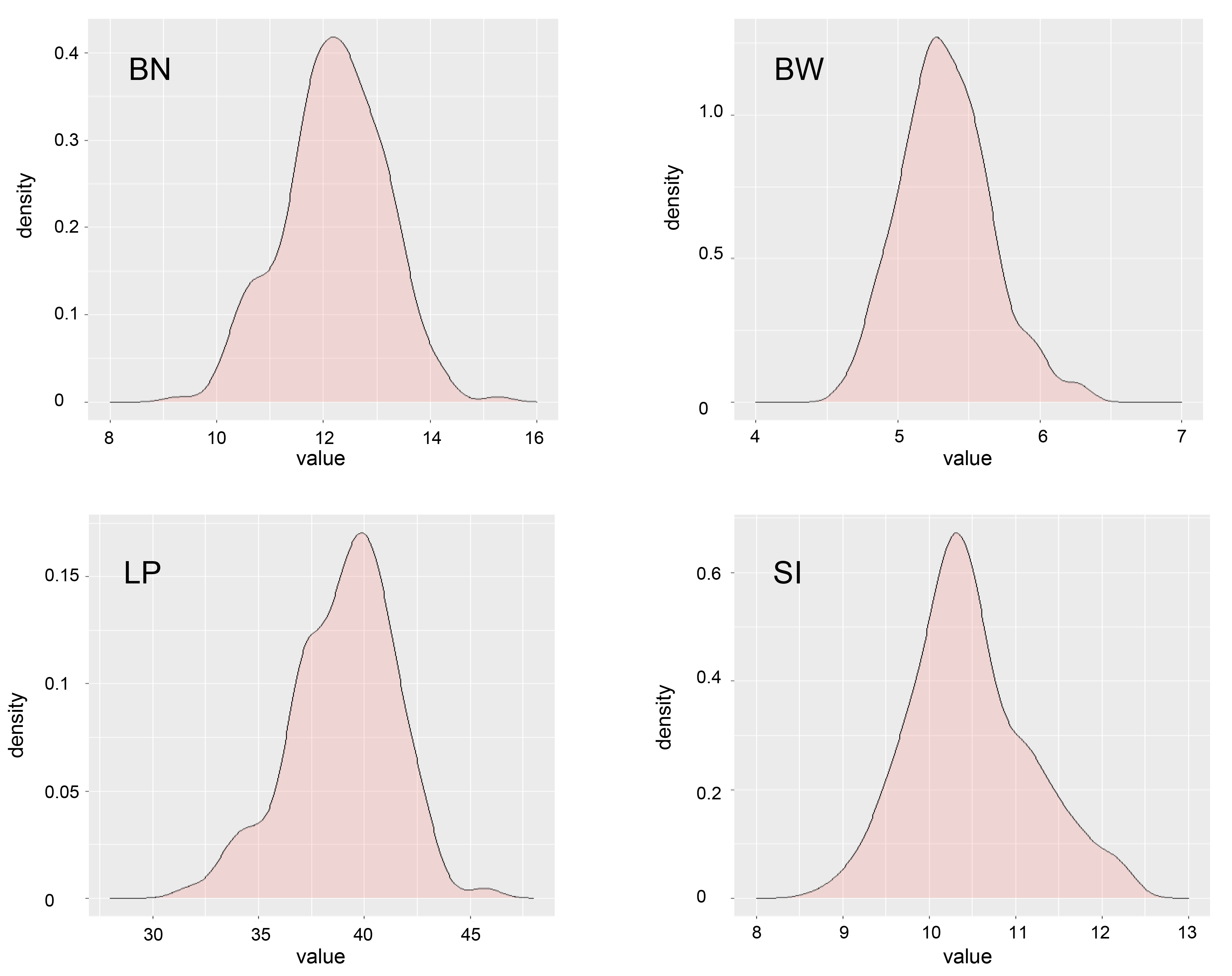

Supplement: Supplementary file 3 — Additional file 3: Figure S1. Density distributions of four yield-related traits in upland cotton natural population. [file 12870_2021_3009_MOESM3_ESM.tif]

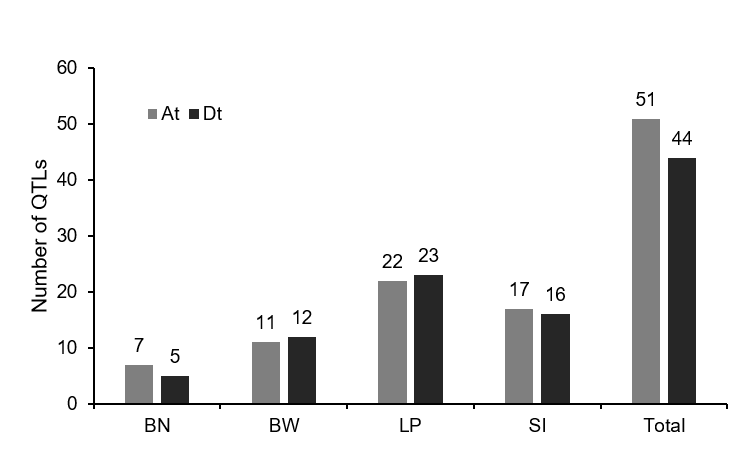

Supplement: Supplementary file 8 — Additional file 8: Figure S2. The number of candidate QTLs associated with the yield-related traits on At and Dt sub-genomes. [file 12870_2021_3009_MOESM8_ESM.tif]

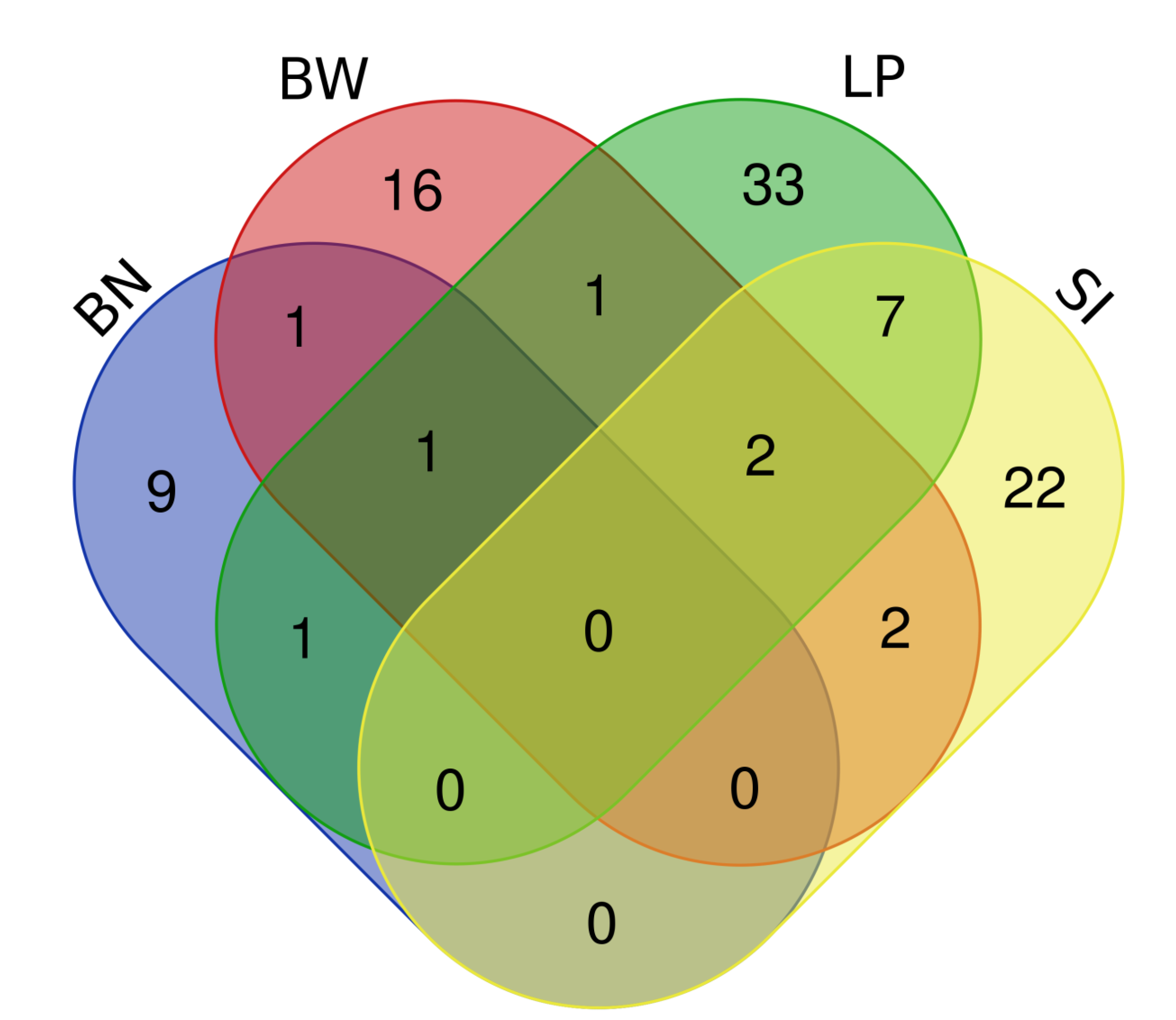

Supplement: Supplementary file 9 — Additional file 9: Figure S3. Venn diagram of QTLs associated with four yield-related traits. [file 12870_2021_3009_MOESM9_ESM.tif]

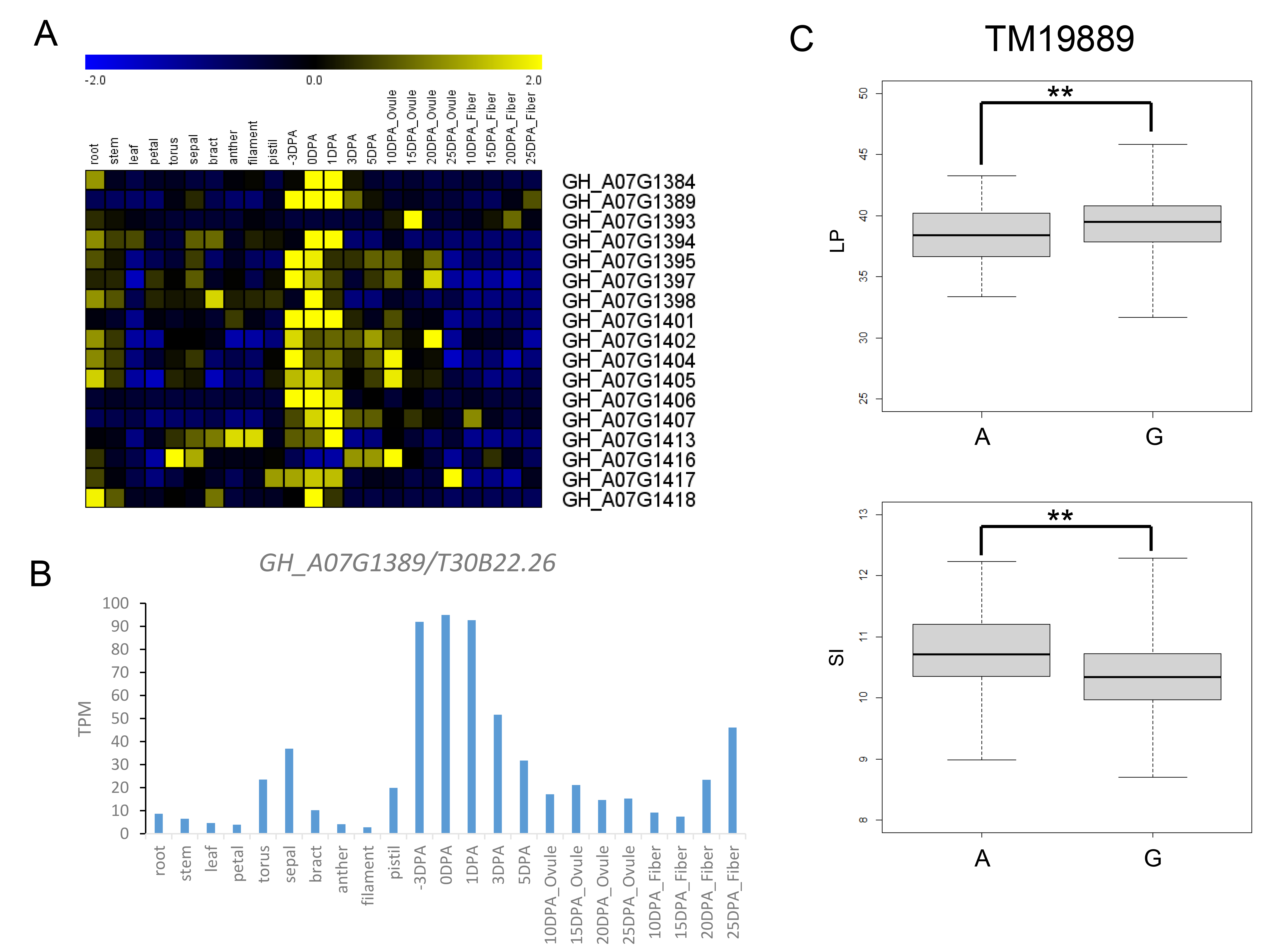

Supplement: Supplementary file 12 — Additional file 12: Figure S4. Candidate genes related to the qlt19 associated with SI and located on chromosome A07. a. Expression heatmap of candidate genes in qlt19. b. The expression pattern of GH_A07G1389 in different tissues. c. Box plots for the phenotypic values of QTN closest to GH_A07G1389. [file 12870_2021_3009_MOESM12_ESM.tif]
